# Diagnosing an atypical site of giant cell arteritis with magnetic resonance angiography: a case report

**DOI:** 10.1186/s13256-016-0971-y

**Published:** 2016-06-23

**Authors:** Boon L. Tan, Jonathan J. Liu, Tuck Y. Yong, Chrismin C. Tan, Jordan Y. Li

**Affiliations:** 1 Department of General Medicine, Flinders Medical Centre, Flinders Drive, Bedford Park, South Australia 5042 Australia; 2 Flinders Private Hospital, Flinders Dr, Bedford Park, SA 5042 Australia; 3 Department of Medical Imaging, Flinders Medical Centre, Flinders Drive, Bedford Park, South Australia 5042 Australia; 4 School of Medicine, Flinders University, Bedford Park, South Australia Australia

**Keywords:** Extracranial involvement, Giant cell arteritis, Magnetic resonance angiography, Vasculitis

## Abstract

**Background:**

Giant cell arteritis typically involves the temporal arteries, but can involve other cranial arteries. Temporal artery biopsy is the mainstay for the diagnosis of giant cell arteritis; however, biopsy may be problematic if giant cell arteritis involves other cranial arteries that are inaccessible for sampling. In these situations, magnetic resonance angiography is a useful, non-invasive adjunctive method in the diagnosis of giant cell arteritis. In this case report, we describe a case of giant cell arteritis involving only the occipital artery which was revealed by magnetic resonance angiography.

**Case presentation:**

A 67-year-old Caucasian man was admitted to our hospital with a 4-week history of malaise, fever, and mild occipital headaches. There were no other positive findings on physical examination. Laboratory studies were remarkable for normocytic anemia, raised inflammatory markers, and mildly deranged liver function tests. To exclude intracranial pathology, he underwent a cranial magnetic resonance imaging with gadolinium, which demonstrated a thickened wall and mural enhancement of his right occipital artery, consistent with giant cell arteritis. His temporal arteries were normal. His occipital arteries were not accessible for biopsy and he was commenced on high-dose prednisolone (60 mg daily). His symptoms resolved completely after a week of glucocorticoid steroid treatment and he was well on 5 mg of prednisolone once a day on follow-up.

**Conclusion:**

While magnetic resonance angiography may not replace the need for biopsy, it may have a diagnostic role in suspected giant cell arteritis, such as when the involved arteries are inaccessible for biopsy.

## Background

Giant cell arteritis (GCA) is a chronic inflammatory disorder characterized by granulomatous infiltrates affecting the large and medium-sized vessels. It is closely related to polymyalgia rheumatica (PMR) [1]. GCA occurs predominantly in females, with a mean age at diagnosis of 72 years [2] and is the most common chronic vasculitis of medium and large arteries in populations with predominantly Northern European ancestry. The diagnostic criteria for GCA according to American College of Rheumatology (ACR), require the fulfilment of at least three of the following five criteria: (i) age more than 50 years, (ii) new headache, (iii) superficial temporal artery tenderness or decreased pulsation, (iv) elevated erythrocyte sedimentation rate (ESR) of more than 50 mm/hour using the Westergren method, and (v) abnormal findings on temporal artery biopsy [3]. GCA can be a diagnostic challenge for physicians due to the wide variation of presentations, false negative histology, absence of potential biopsy sites, or lack of cranial symptoms. Furthermore, cranial arteries other than the temporal artery are involved in more than half of GCA cases [1]. As a result, symptoms are often atypical and may include fever, general malaise and weight loss. We report a case of GCA with only right occipital artery involvement diagnosed by magnetic resonance angiography (MRA).

## Case presentation

A 67-year-old Caucasian man presented with a 4-week history of general malaise, mild occipital headache, and fever. He has a past medical history of hypercholesterolemia and a stable right acoustic neuroma under observation. He described no visual disturbance, jaw claudication, arthralgia, or weight loss.

On physical examination, he was febrile with temperature of 38 °C. He had no active synovitis or rash. His temporal arteries were not palpable; a cranial nerve and funduscopic examination was normal. There was no lymphadenopathy or hepatosplenomegaly. The rest of the physical examination was unremarkable.

Laboratory investigations showed mild normocytic anemia, raised inflammatory markers, and mild deranged liver function test (Table 1). Renal function and electrolytes were within normal reference ranges. Multiple blood and urine cultures were negative. Further investigations including vasculitic screen, complete autoimmune screen, infective viral screen, and malignant screen were either negative or in the normal range (Table 1). Computed tomography (CT) of his neck, thorax, abdomen and pelvis were negative for lymphadenopathy, mass, abscess, and infective focus. A bone morrow biopsy and aspirate showed mild reactive plasmacytosis and the culture was negative for tuberculosis.

**Table 1:** Laboratory, serological, and immunological investigation results

Variables	Results	Normal reference range
Hemoglobin	116 g/L	135–175 g/L
Red blood cells	3.95×10^12^ L	4.50–6.00×10^12^ L
Mean cell volume	91.6 fl	80.0–98.0 fl
Platelet count	484×10^9^/L	150–450×10^9^ L
White cell count	6.61×10^9^/L	4.00–11.0×10^9^/L
Neutrophils	4.83×10^9^/L	1.80–7.50×10^9^/L
Gamma-glutamyl transpeptidase	189 U/L	<60 U/L
Alkaline phosphatase	172 U/L	30–110 U/L
Alanine aminotransferase	165 U/L	<55 U/L
Aspartate aminotransferase	65 U/L	<45 U/L
Lactate dehydrogenase	167U/L	110–230 U/L
Sodium	139 mmol/L	137–145 mmol/L
Potassium	4.2 mmol/L	3.5–4.9 mmol/L
Urea	4.0 mmol/L	2.7–8.0 mmol/L
Creatinine	53 μmol/L	50–120 μmol/L
Erythrocyte sedimentation rate (ESR)	96 mm/h	1–13 mm/h
C-reactive protein (CRP)	400 mg/L	<10 mg/L
Antinuclear antibody	Negative	
Extractable nuclear antibody	Negative	
Anti-neutrophil cytoplasmic	Negative	
Hepatitis B and C serology	Negative	
Liver, kidney mitochondrial antibody	Negative	
Smooth muscle antibody	Negative	
Anti-mitochondrial antibody	Negative	
Serum electrophoresis	No paraprotein	

**Fig. 1:**
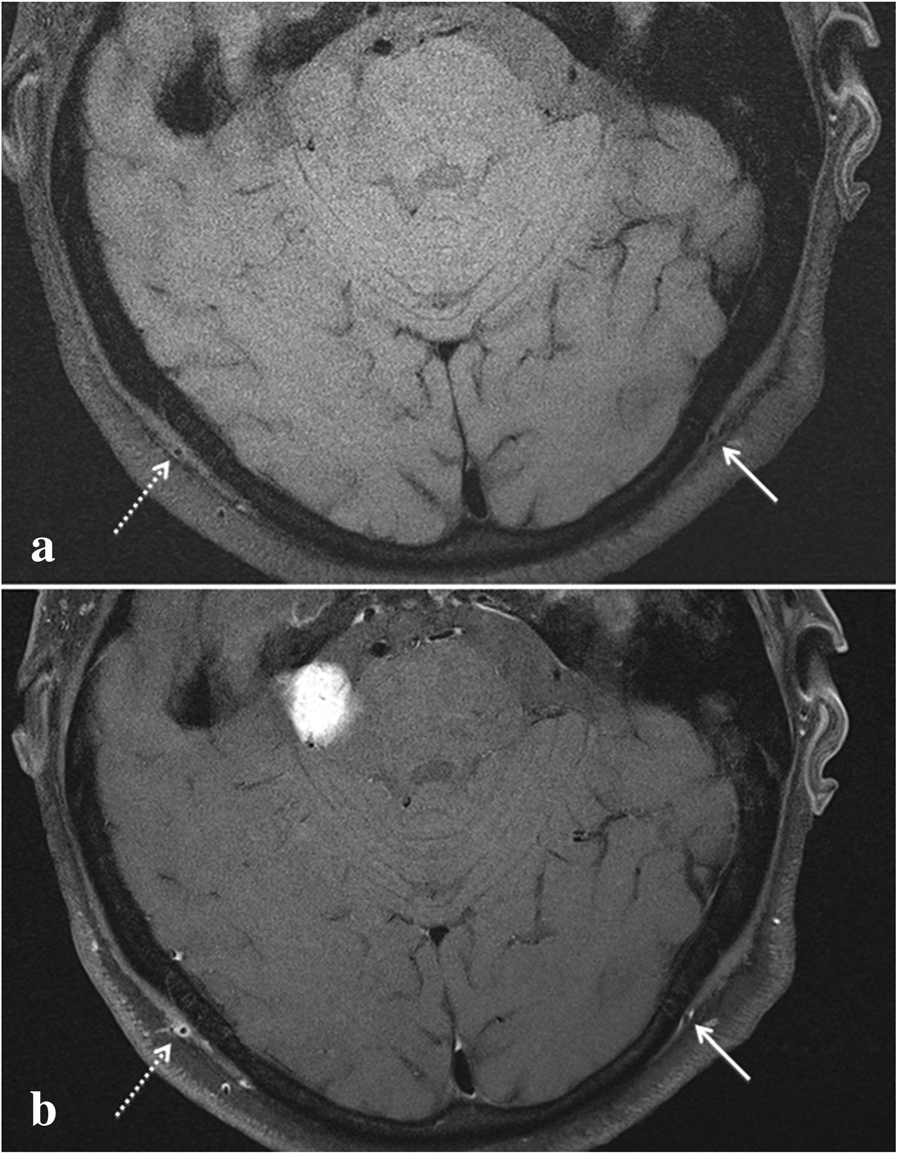
High-resolution T1 fat-saturated magnetic resonance sequences before (**a**) and after (**b**) intravenous gadolinium injection demonstrates mural thickening and enhancement in the right occipital artery (*dashed arrow*). *Solid arrow* denotes the less-affected left occipital artery

In the setting of fever, headache, and raised inflammatory markers, our patient underwent a cranial 3.0 tesla (3T) magnetic resonance imaging (MRI) with gadolinium, which showed a thickened wall and mural enhancement of his right occipital artery consistent with GCA (Fig. 1). His temporal arteries were normal bilaterally. After consultation with vascular surgeons, there was no potential biopsy site to enable histopathological confirmation. A diagnosis of GCA was considered and he was started on oral prednisolone (60 mg daily). He became afebrile on the second day of treatment and all symptoms resolved within 1 week. On follow-up, he has been well and the prednisolone dose has been gradually tapered down to 5 mg daily. Six weeks after the glucocorticoid steroid treatment, he underwent an 18-flurodeoxyglucose (^18^FDG) positron emission tomography (PET) scan, which revealed complete resolution of occipital GCA and no evidence of other vasculitis.

## Discussion

Other cranial arteries can be involved in GCA apart from the temporal arteries [4]. Branches of the external carotid artery, including the posterior ciliary arteries that supply the optic nerve and primary and secondary branches of the aorta are often affected. GCA of the lower extremity arteries and mesenteric arteries is extremely rare, while intracranial arteries are essentially spared [5]. The diagnosis of GCA is usually confirmed by temporal artery biopsy with histopathology demonstrating panarteritis with mononuclear infiltrates penetrating all layers of the arterial wall [1]. However, this can pose a problem in cases where the temporal artery is spared. Furthermore, the inflammation of the temporal artery in GCA can be segmental making the diagnosis of GCA challenging even when the affected artery is accessible for biopsy. An estimate of the actual sensitivity of unilateral temporal artery biopsy is between 69 and 95 % [6]. As a result, there is a growing interest in using radiological imaging in the investigation of patients with suspected GCA [7].

High resolution MRI with gadolinium is a useful non-invasive adjunctive method in diagnosis of GCA. High-resolution MRI in particular allows detailed assessment of the major superficial cranial arteries, and administration of gadolinium allowing detection of arterial wall thickening and periadventitial enhancement suggestive of mural inflammation. These abnormalities are likely to represent the early vascular inflammation that precedes the development of luminal changes and can be used as a guide to estimating disease activity [8]. Single-center and multi-center trials evaluating MRI of the superficial cranial arteries (superficial temporal and occipital arteries) have yielded an overall sensitivity of between 78 and 89 % and specificity between 90 and 92 % for the diagnosis of GCA [7, 9]. A further role for MRI in following up of GCA response to treatment and monitoring for recurrent disease activity has also been proposed [10].

One case report and a small study have suggested that high resolution contrast-enhanced, black-blood T1-weighted images with fat suppression might be useful for the diagnosis of intracranial arteritis [11, 12]. This may allow invasive procedures to be replaced, such as conventional angiography and/or brain biopsies. However, the role of black-blood T1-weighted images in diagnosing extracranial arteritis has not been evaluated. Further studies are needed to confirm these initial promising results.

Conventional angiography and CT angiography can also document large vessel involvement in GCA [13], although the risks associated with radiation exposure and iodinated contrast media may limit scanning in patients who are allergic to contrast or have renal impairment. Sonographic features such as a hypoechoic periluminal halo have been shown to have a high specificity for GCA on meta-analysis, but its sensitivity and specificity values have not been compared directly with MRI [14, 15]. More advanced disease may be seen sonographically as vascular stenosis or occlusion. Recent literature identifies ^18^FDG PET scans as well suited for the demonstration of GCA in arteries exceeding 4 mm in diameter [16]. While not currently felt suitable to replace the need for biopsy, a potential role has been suggested for MRA or ^18^FDG PET scan in the diagnosis of atypical GCA, where conventional clinical features and temporal artery biopsy are non-contributory, or for follow-up after corticosteroid therapy.

## Conclusions

In summary, atypical cases of GCA such as that presented here pose a diagnostic dilemma to the clinician due to the variable and nonspecific nature of patient presentation. Although clinical features may be suggestive, inability to obtain histopathological confirmation can limit diagnostic certainty. The increasing sensitivity and specificity of high-resolution MRI not only allows more confident diagnosis of these atypical cases but may even play a role in monitoring for response to treatment or recurrent disease.
